# Case report: Complete response of an anaplastic thyroid carcinoma patient with *NRAS* Q61R/*BRAF* D594N mutations to the triplet of dabrafenib, trametinib and PD-1 antibody

**DOI:** 10.3389/fimmu.2023.1178682

**Published:** 2023-04-14

**Authors:** Lin Gui, Yiming Zhu, Xiaomo Li, Xiaohui He, Tonghui Ma, Yi Cai, Shaoyan Liu

**Affiliations:** ^1^Department of Medical Oncology, National Cancer Center/National Clinical Research Center for Cancer/Cancer Hospital, Chinese Academy of Medical Sciences & Peking Union Medical College, Beijing Key Laboratory of Clinical Study on Anticancer Molecular Targeted Drugs, Beijing, China; ^2^Department of Head and Neck Surgical Oncology, National Cancer Center/National Clinical Research Center for Cancer/Cancer Hospital, Chinese Academy of Medical Sciences & Peking Union Medical College, Beijing, China; ^3^Genetron Health (Beijing) Technology, Co. Ltd, Beijing, China; ^4^Independent Researcher, Ellicott City, Maryland, MD, United States

**Keywords:** anaplastic thyroid carcinoma (ATC), BRAF non-V600E, NRAS, combination immunotherapy and targeted therapy, case report

## Abstract

Anaplastic thyroid carcinoma, *BRAF* non-V600, *NRAS*, combination immunotherapy and targeted therapy, case report. Anaplastic thyroid carcinoma (ATC) is a rare type of thyroid cancer with a mortality rate near 100%. *BRAF* V600 and *NRAS* mutations are the most common drivers of ATC. While patients with *BRAF* V600-mutated ATC can be treated with BRAF-targeted therapy, there is no effective treatment for ATC driven by *NRAS* or non-V600 *BRAF* mutations. For patients with untargetable driver mutations, immunotherapy provides an alternative treatment option. Here, we present a metastatic ATC patient with PD-L1 positive (tumor proportion score of 60%) tumor and *NRAS* Q61R/*BRAF* D594N mutations, who progressed on PD-1 antibody sintilimab plus angiogenesis inhibitor anlotinib. The class 3 BRAF mutant D594N is sensitive to the inhibition of MEK inhibitor trametinib, and its oncogenic activity also depends on CRAF, which can be inhibited by BRAF inhibitor dabrafenib. For these reasons, the patient received a salvage treatment regime of dabrafenib, trametinib, and sintilimab, which resulted in a complete pathological response. To our best knowledge, this is the first report of successful treatment of ATC patients with concurrent *NRAS*/*BRAF* non-V600 mutations with the combination of immunotherapy and targeted therapy. Further investigation is required to decipher the mechanism by which the combination of dabrafenib/trametinib with PD-1 antibody overcomes initial immunotherapy resistance likely mediated by concurrent *BRAF* and *NRAS* mutations.

## Background

Anaplastic thyroid carcinoma (ATC) is one of the most aggressive solid tumors with a disease-specific mortality rate near 100% (1). Genomic profiling studies revealed that the major drivers of ATC are *TP53*, *TERT*, *BRAF*, and *NRAS* mutations (2). While ATC patients with *BRAF* V600 mutation are eligible for BRAF-targeted therapy, there is no effective treatment for *NRAS*-mutated ATC patients (1).

In the past decade, cancer immunotherapy has shifted the paradigm of cancer treatment. Based on the results of the KEYNOTE-158 trial, FDA approved PD-1 antibody pembrolizumab as a treatment option for patients with TMB-H (≥ 10 mut/Mb) solid tumors, which was also endorsed by the NCCN thyroid cancer guideline for ATC treatment (1). Of note, the KETNOTE-158 trial only included two patients with thyroid cancer. Recently, an investigational PD-1 antibody spartalizumab showed promising efficacy in a phase 2 trial of ATC, in which spartalizumab achieved an overall response rate (ORR) of 29% and 35% in the PD-L1 positive and high PD-L1 (≥ 50%) subgroups, respectively (3). Responses were seen in *BRAF* wild-type and *BRAF*-mutant patients. These results indicated that PD-1 antibody might be a treatment option for ATC patients with high PD-L1 expression, irrespective of their *BRAF* mutation status.

Both immune checkpoint blockade (ICB) and BRAF-targeted therapies have been approved for the treatment of *BRAF* V600-mutant melanoma (4). Of note, abnormal activation of the MAPK signaling can result in tumor-intrinsic resistance to ICB through the modulation of tumor microenvironment (TME) (5). The triplet combination of BRAF inhibitor (BRAFi), MEK inhibitor (MEKi), and PD-1/L1 antibodies have been tested in clinical trials of *BRAF* V600-mutant melanoma (6–8). Although the triplet regimes did not improve the response rate compared with the doublets, they improved the duration of response. Furthermore, two triplets improved progression-free survival (PFS) in the IMspire150 and KEYNOTE-022 trials (6, 7). Therefore, the NCCN guideline for cutaneous melanoma recommended two triplets regimes (vemurafenib/cobimetinib/atezolizumab and dabrafenib/trametinib/pembrolizumab) as first-line therapy options for unresectable or metastatic melanoma patients with *BRAF* V600 mutation (4).

**Figure 1:**
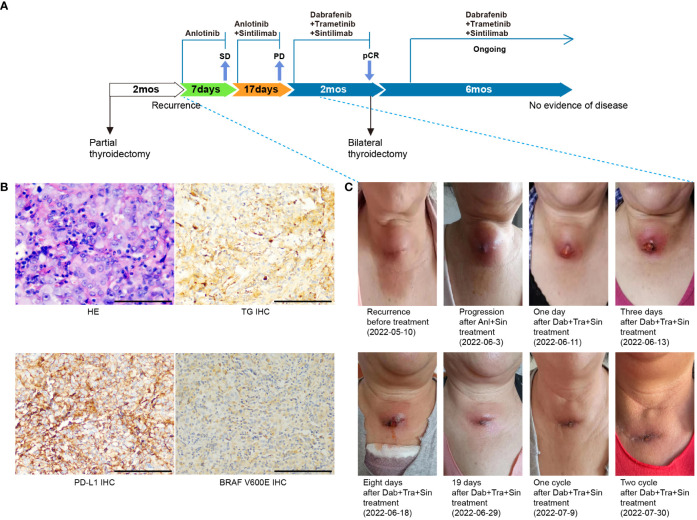
Case summary. **(A)** Summary of disease course and treatment procedure. pCR, pathological complete response; SD, stable disease; PD, progressive disease; mo, months. **(B)** H&E, TG, BRAF V600E and PD-L1 staining of the primary tumor. Scale bars: 50 µm. H&E: hematoxylin and eosin. **(C)** Representative images showing disease recurrence/progression on sintilimab plus anlotonib, and the patient’s response to the triplet regime of sintilimab, dabrafenib, and trametinib.

According to the mechanisms of activation, BRAF mutants can be classified into three groups (9). Class 1 and 2 mutants function as RAS-independent monomers and dimers, respectively (9). Class 3 mutants have impaired kinase activity, and their oncogenic activity depends on RAS and CRAF (9). Because coexisting *BRAF* mutations synergize with *RAS* mutations in the amplification of downstream MAPK signaling, the treatment of solid tumors with coexisting *BRAF/RAS* mutations is more challenging than those with *BRAF* or *RAS* mutations alone. Here, we presented a PD-L1-positive metastatic ATC patient with coexisting *NRAS* and class 3 *BRAF* mutations. After progression on PD-1 antibody sintilimab plus an angiogenesis inhibitor anlotinib, she achieved a pathological complete response with the triplet regime of BRAFi dabrafenib, MEKi trametinib, and a domestic PD-1 antibody sintilimab (**Figure 1A**).

## Case presentation

**Figure 2:**
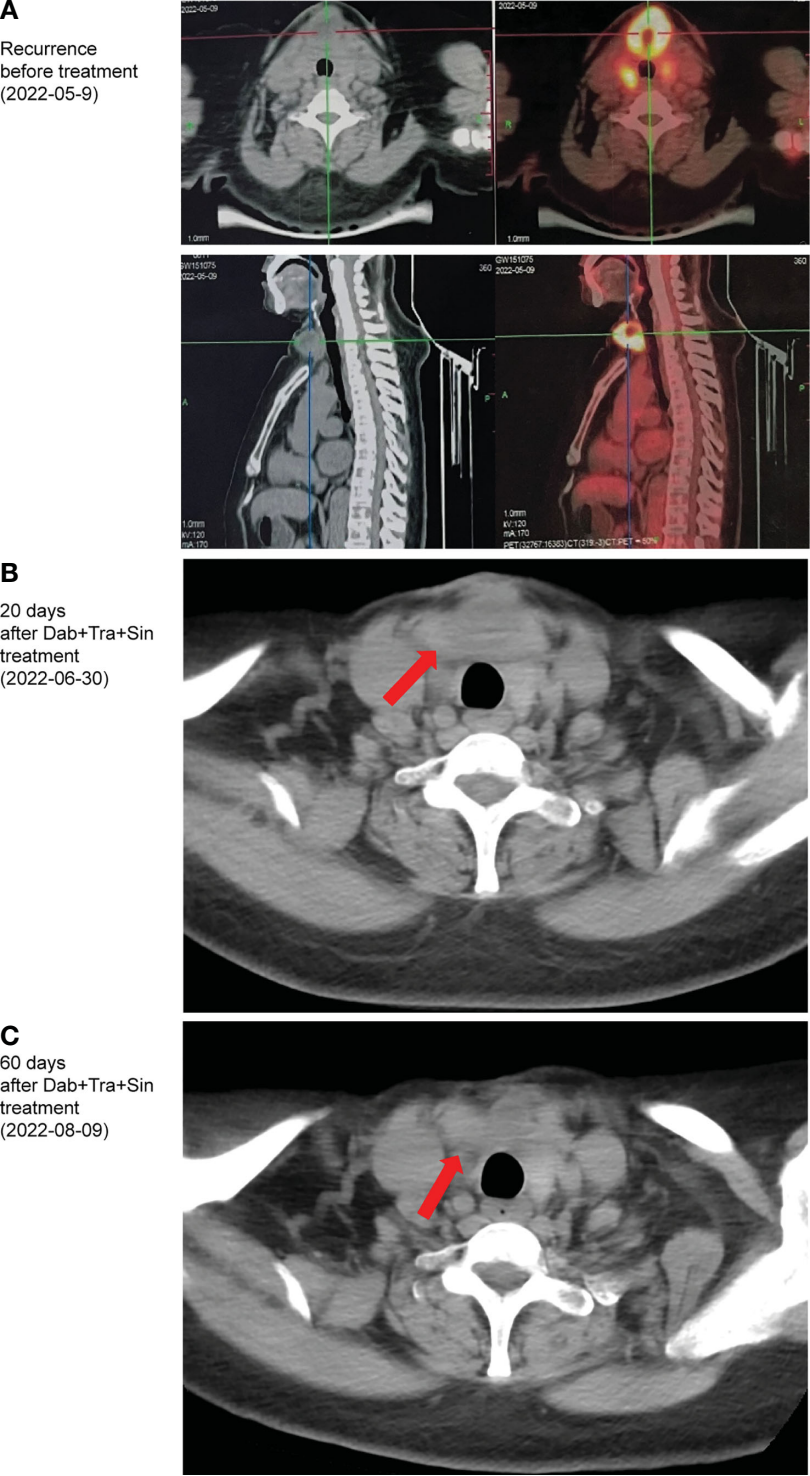
Computed tomography images showing the patient’ recurrence **(A)**, as well as response to the triplet regime of sintilimab, dabrafenib, and trametinib after 20 **(B)**, 60 **(C)** days of treatment.

A 61-year-old female presented with a rapidly enlarging neck mass in November 2021. Contrast-enhanced computed tomography (CT) revealed a 1.9×1.8 cm nodule and a 3.2×2.6 cm nodule in the left and right thyroid lobes, respectively. She underwent surgical resection of partial thyroid right lobe and thyroid left lobe mass on Jan 25^th^, 2022. Postoperative histopathological examination confirmed multifocal anaplastic thyroid carcinoma with areas of necrosis and calcifications in the right lobe, and nodular goiter in the left. Immunohistochemical (IHC) staining was positive for Ki-67 (80%), Vimentin, TTF-1, P53, Cyclin D1, Pax-8, TG (weak, 1+), BRAF V600E (weak, 1+), and negative for AE1/AE3, CD56, Calcitonin, LCA, and HMB45 (**Figure 1B**). Two months later, a progressively enlarging mass appeared in her right neck, indicating disease progression (**Figure 1A**). PET-CT imaging revealed multiple hypoechoic nodules in cervical region VI, with the largest located in the center of 4.0×2.8 cm (**Figures 1C**, **2A**). The patient was then referred to our hospital. She had a poor Eastern Cooperative Oncology Group (ECOG) performance status of 2, difficulty in breathing, and choking with deglutition.

Because radical surgery was not suitable, the patient was treated with anlotinib (10mg, QD, 2 weeks on/1 week off), a multi-targeted tyrosine kinase inhibitor targeting tumor angiogenesis and proliferation from May 13^th^. The lesion soon shrank by about 20%, and the patient’s skin color returned to normal. Genetic testing of the resected tumor tissue with a multi-gene next-generation sequencing (NGS) panel (Onco Panscan™, Genetron Health) revealed a tumor mutational burden of 1.41 mutations per megabase (mut/Mb), microsatellite status stable (MSS), *TERT* promoter mutation, an *NRAS* Q61R mutation with a variant allele fraction (VAF) of 10.6%, and a *BRAF* D594N mutation with a VAF of 12.6% (**Table 1**). PD-L1 IHC assay (PD-L1 IHC 22C3 pharmDx assay, Agilent Technologies, Carpinteria, CA, USA) showed a TPS of 60% and a CPS of 80 (**Figure 1B**). We previously reported the successful treatment of a PD-L1-positive, *NRAS* Q61R-mutated metastatic ATC patient with sintilimab plus an angiogenesis inhibitor anlotinib (10). Given the similarity of these two ATC patients, sintilimab (200 mg, Q3W) was added on May 21^st^, 2022. Unfortunately, disease progression occurred, including neck mass enlargement to 4.4×3.0 cm, the reappearance of breathing difficulty, and choking with deglutition. And this doublet regime was left off after one cycle (**Figures 1A, C**).

**Table 1:** Summary of NGS analysis.

Mutation Type	Gene	Nucleotide change	Amino acid change	Mutation effect	VAF (%)
Somatic	*BRAF*	c.1782T>G	p.Asp594Glu	Missense	12.6
	*NRAS*	c.182A>G	p.Gln61Arg	Missense	10.6
	*CDK8*	c.524G>C	p.G175A	Missense	10.8
	*TERT*	c.-124C>T	–	Promoter	33.6
MSS					
TMB low (1.41mutations/MB)					

VAF, variant allele frequency; MSI, microsatellite instability; MSS, microsatellite stable; TMB, tumor mutational burden.

MAPK signaling amplification driven by class 3 BRAF mutant D594N is sensitive to the inhibition of trametinib (9). Furthermore, the oncogenic activity of class 3 BRAF mutants depends on CRAF (11), which can be inhibited with dabrafenib (12). Of note, the efficacy of dabrafenib plus trametinib has not been established in *NRAS*-driven solid tumors. Given the coexistence of *NRAS* Q61R and *BRAF* D594N mutations, the patient was then treated with a salvage regime consisting of dabrafenib (75 mg, BID), trametinib (2 mg, QD), and sintilimab (200mg, Q3W) from Jun 10^th^, 2022. After nine days, regression of all metastases in the bilateral neck was noted, and breathing/swallowing returned to normal (**Figures 1A, C**). After one cycle, the neck mass continued to shrink, and the ulcerated area was healed (**Figures 1C**, **2B**). Fever (39.5°C) was the only adverse event observed during this triplet regime treatment. Her temperature returned to normal within 24 hours of taking off dabrafenib. The triplet regime was resumed after her temperature remained normal for 24 hours, and no subsequent fever occurred. After two cycles (six weeks) of treatment, radical surgery was evaluated as feasible (**Figures 1C**, **2C**). The patient discontinued dabrafenib and trametinib on Aug 11^th^, 2022. And bilateral residual thyroidectomy plus neck lymph node dissection were performed the next day. Postoperative pathology indicated that she had a pathological complete response (pCR). Given the high risk of ATC patients with concomitant *BRAF*/*RAS* and *TERT* mutations (13), she continued on the triplet regime from Sep 20^th^ and remained in remission with an excellent quality of life until the last follow-up in March 2023 (**Figure 1A**).

## Discussion

The three major histological types of thyroid cancers are differentiated thyroid carcinoma (DTC), medullary thyroid carcinoma (MTC), and anaplastic thyroid carcinoma (ATC) (1). DTC represents more than 95% of thyroid cancer cases and has a very good prognosis. In contrast, ATC patients have a poor prognosis with a historical median overall survival (OS) of four months (14). ATC responds poorly to conventional thyroid cancer treatment options including surgery, radiation therapy, chemotherapy, and radioactive iodine (RAI) therapy (1). The NCCN guideline for thyroid carcinoma recommended that ATC patients with locally resectable disease can be treated with multimodal therapy, and those with actionable mutations (*BRAF*, *NTRK*, *ALK*, *RET*, MSI, dMMR, TMB-H) can be treated with targeted therapy or immunotherapy (1).

Recent genomic profiling studies revealed that activating *BRAF* and *RAS* mutations are major drivers of thyroid cancer. The TCGA study characterized the landscape of papillary thyroid carcinoma (PTC), the most common type of thyroid cancer (15). Among 496 PTC patients, 60% carried *BRAF* mutations, and 13% had *RAS* mutations. Compared to PTC, genomic studies of large ATC cohorts are rare. In one study of 126 ATC patients, 45% had *BRAF* alterations (all V600E except for 1 deletion), and 24% had *RAS* mutations (13). In another ATC cohort (n = 196), 41% had *BRAF* mutations and 27% had *RAS* mutations (2). Interestingly, the latter study also observed non-V600 *BRAF* mutations in PTC, one precursor of ATC. These rare *BRAF* alterations include *BRAF* fusions, K601E, G469A, V600_K601delinsE, V600_K601>D, V600_W604>R, and V600_S605>D. Of note, *BRAF* V600E and *RAS* mutations are mutually exclusive in these thyroid cancer cohorts.

Abnormal activation of the RAF-RAS-MAPK signaling pathway is identified in more than 30% of human cancers (16). BRAF is an established therapeutic target in colorectal cancer (CRC), non-small cell lung cancer (NSCLC), melanoma, and ATC (17). Recently, FDA granted accelerated approval of dabrafenib in combination with trametinib for the treatment of almost all *BRAF* V600-mutated solid tumors. In contrast, there is no effective therapy for *NRAS*-mutated solid tumors. Furthermore, coexisting *NRAS* mutations can result in resistance to dabrafenib/trametinib (18, 19). Therefore, the treatment of solid tumors driven by *NRAS* mutation alone or in combination with *BRAF* mutations represents a clinical challenge.

Recent pieces of evidence indicated that immunotherapy could be an effective treatment option for ATC. In a single-center study, PD-1 antibody pembrolizumab or nivolumab achieved an overall response rate (ORR) of 16% in 13 advanced or metastatic ATC patients, including seven patients with *BRAF* V600E mutations (20). And responses were ongoing in four individuals. Similarly, in a phase 2 trial of advanced/metastatic ATC (n = 42), an investigational PD-1 antibody spartalizumab achieved an overall ORR of 19%, including three complete responses (CRs) and five partial responses (PRs) (3).

Given the poor prognosis and limited therapeutic options of ATC, several multitargeted tyrosine kinase inhibitors (TKIs) including sorafenib, sunitinib, imatinib, and pazopanib have been evaluated in clinical trials with unsatisfactory results. Anlotinib is a novel multitarget TKI which has been approved in China for the treatment of medullary thyroid cancer (MTC), (NSCLC), small cell lung cancer (SCLC), soft tissue sarcoma (STS) and radioactive iodine-refractory differentiated thyroid cancer (RAIR-DTC) (21, 22). In a single-arm phase 2 trial, neoadjuvant anlotinib therapy achieved an ORR of 76.9% in patients with advanced thyroid cancer (23). As most patients treated with TKI will develop resistance, the combination of PD-1 antibodies with TKIs has been proposed as a strategy to overcome TKI resistance in cancer patients. In a retrospective study at the MD Anderson Cancer Center, the addition of pembrolizumab to TKIs resulted in a best overall response (BOR) of 42% in ATC patients who progressed on TKIs (24). Sintilimab is a PD-1 antibody approved in China for the treatment of NSCLC, Hodgin’s lymphoma, hepatocellular carcinoma, and esophageal squamous cell carcinoma (ESCC) (25). Recently, the combination of sintilimab and anlotinib has shown promising antitumor activity and tolerable safety profiles in cervical cancer, endometrial cancer, hepatocellular carcinoma, and biliary tract cancer (26–29).

Previously, we reported that sintilimab plus anlotinib achieved a remarkable response in a metastatic *NRAS-*mutated ATC patient with high PD-L1 expression (10). In this study, our patient briefly responded to anlotinib but quickly developed resistance to sintilimab plus anlotinib. We suspected that the distinct responses to sintilimab plus anlotinib in these two PD-L1-positive, *NRAS*-mutated ATC patients could be mediated by the class 3 *BRAF* mutation D594N, which synergizes with *RAS* mutations in the amplification of downstream MAPK signaling (9). The oncogenic activity of class 3 BRAF mutants depends on the kinase activity of CRAF (9), which can be inhibited by BRAFi dabrafenib. Furthermore, class 3 BRAF mutants are sensitive to the inhibition of MEKi trametinib (9). Although the antitumor activity of MAPK-targeted therapy has not been established in *NRAS*-mutated solid tumors, this approach could counteract the synergistic effect of coexisting *NRAS*/*BRAF* mutations and render the tumor sensitive to immunotherapy.

Activation of the MAPK pathway plays a key role in tumor-intrinsic resistance to immune checkpoint blockade (30). In patients with triple-negative breast cancer, activation of the RAS-MAPK pathway was associated with reduced tumor-infiltrating lymphocytes (TILs) (31). However, trametinib treatment upregulated the expression of PD-L1 and MHC-I/II in mouse mammary tumor-derived cell lines *in vitro* and *in vivo*. Similarly, in *BRAF* V600-mutant melanoma, hyperactivated MAPK signaling inhibits T cell infiltration through the production of VEGF, which was reversed by the administration of a BRAFi (32). Other studies showed that the addition of dabrafenib/trametinib to immune checkpoint blockade (ICB) resulted in higher antitumor activity than ICB alone (33, 34). These preclinical and clinical studies led to the hypothesis that the combination of immunotherapy and MAPK-targeted therapy might provide better clinical benefits than each alone. The efficacy of BRAFi/MEKi plus PD-1/PD-L1 antibody triplets in *BRAF*-mutated solid tumors has been tested in three pivotal trials of melanoma (IMspire150, KEYNOTE-022, COMBI-i). Based on the results of the IMspire150 trial, FDA approved the combination of BRAFi vemurafenib, MEKi cobimetinib, and PD-L1 antibody atezolizumab for the treatment of *BRAF* V600-mutated melanoma. Because class 3 BRAF mutants were resistant to vemurafenib but sensitive to trametinib, we selected the dabrafenib/trametinib doublet to combine with sintilimab as the salvage therapy for our patient, which achieved a complete pathological response.

In addition to triplet therapy, optimal sequencing of immunotherapy and targeted therapy represents another strategy to improve outcomes for patients with *BRAF*-mutated solid tumors. In murine models of *BRAF*/*NRAS*-driven melanoma, anti-PD-1/L1 antibody lead-in before MAPK inhibitor combination optimized antitumor activity by promoting T cell clonal expansion and macrophage polarization (35). Consistently, results of two trials (DREAMseq and SECOMBIT) showed that nivolumab/ipilimumab followed by BRAFi/MEKi doublets led to superior overall survival in patients with advanced *BRAF*-mutant melanoma when compared with the opposite treatment sequence (36, 37). Similarly, data from the phase 3 NEMO trial revealed that previous immunotherapy led to better clinical benefits of MEKi binimetinib in patients with *NRAS*-mutant melanoma (38). While these results were obtained in patients with *BRAF*/*NRAS*-mutant melanoma, we can not rule out the possibility that a brief preceding immunotherapy of our patient may also contribute to her response to the BRAFi/MEKi/PD-1 antibody triplet regime.

Given this patient’s remarkable response, further investigation is required to explore the potential of the BRAFi/MEKi/PD-1 antibody triplet in patients with *NRAS-* or *BRAF*-mutated solid tumors refractory to immunotherapy and *BRAF*-targeted therapy. Furthermore, clinical trials of sequential immunotherapy and targeted therapy in ATC should be pursued.

## Data availability statement

The original contributions presented in the study are included in the article/Supplementary Material. Further inquiries can be directed to the corresponding authors.

## Ethics statement

Written informed consent was obtained from the individual(s) for the publication of any potentially identifiable images or data included in this article.

## Author contributions

Concept and design: LG, SL, YZ, XH. Acquisition, analysis, and interpretation of data: LG, XL, YC. Drafting of the manuscript: YC, XL. Critical revision of the manuscript for important intellectual content: TM, XH, SL. Technical and material support: LG, XH, YZ. Study supervision: SL. All authors contributed to the article and approved the submitted version.
